# Mid-gestational changes in cervicovaginal fluid cytokine levels in asymptomatic pregnant women are predictive markers of inflammation-associated spontaneous preterm birth

**DOI:** 10.1016/j.jri.2018.01.001

**Published:** 2018-04

**Authors:** Emmanuel Amabebe, David R. Chapman, Victoria L. Stern, Graham Stafford, Dilly O.C. Anumba

**Affiliations:** a Academic Unit of Reproductive and Developmental Medicine, The University of Sheffield, Sheffield, UK; b Integrated BioSciences Group, School of Clinical Dentistry, The University of Sheffield, Sheffield, UK

**Keywords:** PTB, preterm birth, sPTB, spontaneous preterm birth, PTL, preterm labour, CVF, cervicovaginal fluid, FFN, fetal fibronectin, qFFN, quantitative fetal fibronectin, CL, ultrasound cervical length, RANTES, regulated on activation normal T cell expressed and secreted, BMI, body mass index, GTP, gestational time point, CBA, cytometric bead array, PCR, polymerase chain reaction, ROC, receiver operating characteristics curve, rDNA, ribosomal deoxyribonucleic acid, MMP, matrix metalloproteinase, PG, prostaglandin, Preterm birth, Cervicovaginal fluid, Cytokines, RANTES, IL-1β, Fetal fibronectin

## Abstract

**Objectives:**

Perturbation of the choriodecidual space before the onset of spontaneous preterm birth (sPTB) could lead to a concomitant rise in both cervicovaginal fluid (CVF) cytokine and fetal fibronectin (FFN), and assessing the concentrations of both markers could improve the prediction of sPTB (delivery before 37 completed weeks of gestation). Therefore, we prospectively determined mid-trimester changes in CVF cytokine and FFN concentrations, and their predictive capacity for sPTB in asymptomatic pregnant women.

**Study design:**

CVF collected at 20^+0^–22^+6^ weeks (n = 47: Preterm-delivered = 22, Term-delivered = 25) and 26^+0^–28^+6^ weeks (n = 50: Preterm-delivered = 17, Term-delivered = 33) from 63 asymptomatic pregnant women at risk of sPTB were examined. Cytokine and FFN concentrations were determined by multiplexed bead-based immunoassay and 10Q Rapid analysis (Hologic, MA, USA) respectively. The 20^+0^–22^+6^/26^+0^–28^+6^ weeks ratios of cytokines and FFN concentrations were compared between preterm- and term-delivered women using Receiver Operating Characteristics curves to predict sPTB. Also, bacterial 16S rDNA from 64 samples (20^+0^–22^+6^ weeks n = 36, 26^+0^–28^+6^ weeks n = 28) was amplified by polymerase chain reaction to determine associations between vaginal microflora, cytokine and FFN concentrations.

**Results:**

Changes in RANTES and IL-1β concentrations between 20^+0^–22^+6^ and 26^+0^–28^+6^ weeks, expressed as a ratios, were predictive of sPTB, RANTES (AUC = 0.82, CI = 0.62–0.94) more so than IL-1β (AUC = 0.71, CI = 0.53–0.85) and FFN (not predictive). Combining these markers (AUC = 0.83, CI = 0.63–0.95) showed similar predictive capacity as RANTES alone. FFN concentrations at 26^+0^–28^+6^ weeks correlated with IL-1β (*r* = 0.4, *P* = 0.002) and RANTES (*r* = 0.3, *P* = 0.03). In addition, there was increased prevalence of vaginal anaerobes including *Bacteroides*, *Fusobacterium* and *Mobiluncus* between gestational time points in women who experienced sPTB compared to the term women (*P* = 0.0006).

**Conclusions:**

CVF RANTES and IL-1β in mid-trimester of pregnancy correlate with quantitative FFN. The levels of CVF RANTES and IL-1β decline significantly in women who deliver at term unlike women who deliver preterm. This observation suggests that sPTB may be characterised by sustained choriodecidual inflammation and may have clinical value in serial screening for sPTB if confirmed by larger studies.

## Highlights

• Spontaneous preterm birth is associated with elevated CVF RANTES and IL-1β in mid-trimester.
• High CVF RANTES and IL-1β correlate with FFN, pH and increased prevalence of vaginal anaerobes.
• Increasing/elevated CVF RANTES in mid-trimester is highly predictive of spontaneous PTB than IL-1β and FFN.

## 1 Introduction

Ascending genital tract infection due to changes in the vaginal microbiota induces immune responses characterised by the release of inflammatory cytokines and chemokines capable of initiating preterm labour (PTL) and preterm birth (PTB) (Goldenberg et al., 2000; Huang et al., 2014; Jefferson, 2012). Intrauterine infection during gestation and the subsequent inflammatory processes that ensue can disrupt the maternal choriodecidual tissues and trigger matrix remodelling with concomitant leakage of fetal fibronectin (FFN) into the cervicovaginal space (Agrawal and Hirsch, 2012). Cervicovaginal fluid (CVF) quantitative fetal fibronectin (qFFN) is putatively the most widely employed clinical test for predicting PTB in asymptomatic and symptomatic women. However, it is most often employed in assessing women presenting with symptoms of PTL (Abbott et al., 2015; Heng et al., 2015; Stock et al., 2015) as a rule-out test, largely due to its high negative predictive value (Abbott et al., 2015).

Altered cytokine concentrations between gestations may reflect inflammatory processes associated with spontaneous preterm birth (sPTB). Several inflammatory mediators of PTB in asymptomatic and symptomatic pregnant women, including Interleukin (IL) -1, 2, 4, 6, 8, 10, 12 and 17, tumor necrosis factor-alpha (TNF-α), Interferon gamma (INF-γ), RANTES (regulated on activation, normal T cell expressed and secreted), C-reactive protein (CRP), have been investigated in CVF, amniotic fluid and blood (Agrawal and Hirsch, 2012; Chan, 2014; Chandiramani et al., 2012, Goldenberg et al., 2005; Hee, 2011; Vogel et al., 2007; Vrachnis et al., 2012; Witkin et al., 2011). However, none of these markers has been noted to attain clinically applicable predictive utility for PTB in asymptomatic (low and high risk) (Conde-Agudelo et al., 2011; Hee, 2011) as well as symptomatic pregnant women (Hee, 2011); and especially in asymptomatic women at mid-gestation (Gervasi et al., 2012). Regarding these studies, differences in clinical settings and experimental designs (e.g. sample sizes, inclusion/exclusion criteria, gestational age at sampling etc.) have militated against the identification of consistently accurate predictive biomarkers of PTB (Heng et al., 2015). Improving study designs and methodologies, and exploring multiple promising biochemical diagnostic tests may therefore improve the prediction of PTB (Chan, 2014; Georgiou et al., 2015; Heng et al., 2015).

Furthermore, false positive CVF FFN test results contribute to the relatively low sensitivity and positive predictive value of the test, often resulting from factors such as unprotected vaginal intercourse, digital examination, bleeding or contamination with amniotic fluid following ruptured membranes (Heng et al., 2015). Hitherto, no studies have explored combining the predictive utility for sPTB of CVF cytokine concentrations with qFFN in high-risk asymptomatic women.

We hypothesised that perturbation of the choriodecidual space before the onset of sPTB would lead to a concomitant rise in both CVF cytokine and qFFN, and that assessing the concentrations of both markers could improve the prediction of sPTB (spontaneous delivery before 37 completed weeks of gestation). This study explores this hypothesis by investigating the changes in CVF cytokine and FFN concentrations across mid-trimester in asymptomatic high-risk women who subsequently delivered prematurely and those who delivered at term. We determined whether these changes in cytokine concentrations are predictive of sPTB, either alone or in combination with qFFN.

## 2 Materials and methods

### 2.1 Study design

This is a predefined pilot case-control study that was reviewed and approved by the Yorkshire & Humber (Sheffield) Committee of the UK National Research Ethics Service (REC Number 13/YH/0167). All samples were obtained from participants following written informed consent.

#### 2.1.1 Study participants and sample collection

CVF samples were obtained by high-vaginal swabs (HVS) from 63 asymptomatic high-risk pregnant women at 2 mid-gestational time points (GTP): 20^+0^–22^+6^ weeks (GTP1, n = 47: Preterm-delivered = 22, Term-delivered = 25) and 26^+0^–28^+6^ weeks (GTP2, n = 50: Preterm-delivered = 17, Term-delivered = 33). These GTPs, which fall within the gestational window for FFN and cervical length assessment recognised in clinical practice (Abbott et al., 2015; Esplin et al., 2017; Hezelgrave et al., 2016; Hezelgrave et al., 2017; Jwala et al., 2016; Vandermolen et al., 2016), were selected to determine whether early identification of women at greatest risk may improve prevention of sPTB and its complications. Participants were asymptomatic pregnant women at high-risk of PTB on the basis of a previous history of sPTB and/or a short cervix measuring <25 mm on transvaginal ultrasound (in previous or current pregnancy), attending the specialist antenatal clinics at the Jessop Wing Maternity Hospital, Sheffield, UK, between May 2013 and September 2015. Women presenting with symptoms suggestive of threatened PTL, prelabour ruptured membranes or carrying multiple gestations were excluded from the study, as were those with a recent vaginal examination, or evidence of genital tract infection (e.g. bacterial vaginosis, BV), urinary tract infection or abnormal cervical cytology. PTB outcome was defined as spontaneous delivery before 37 completed weeks of gestation.

At presentation and before any intervention was administered, CVF was obtained from the posterior vaginal fornix of each woman by two high vaginal swabs (HVS, sterile Dacron swabs – Deltalab Eurotubo 300263, Fisher Scientific, UK), after passage of a sterile Cusco’s vaginal speculum. One swab was used for the current study and the other for an independent metabolomics examination. For this study, one swab saturated with CVF was immediately processed or stored in −20 °C and then to −80 °C for approximately 3 days pending analysis. The specimens were subsequently processed by washing the CVF off the swab in a clean 1.5 μl microfuge tube containing 400 μl isotonic Phosphate Buffered Saline (PBS). This was done by vortexing the cut end of the swab in PBS solution for 5 min. From the solution, 250 μl was aspirated and transferred into a fresh 1.5 μl microfuge tube for 16S ribosomal DNA extraction to determine the microbial composition of the vaginal environment, while 50 μl was transferred into a separate tube for determination of cytokine concentration. The remnants (i.e. swabs in 100 μl solution) were stored at −80 °C as part of a growing biorepository.

### 2.2 CVF quantitative fetal fibronectin, vaginal pH and cervical length measurements

Study participants also had vaginal swab specimens assessed for qFFN and pH while cervical length was measured by transvaginal ultrasonography. FFN concentrations were quantified using the 10Q Rapid FFN analyser (Hologic, MA, USA), while vaginal pH was determined by a narrow range pH paper (pH-Fix 3.6–6.1, Machery-Nagel, Düren, Germany) (Jespers et al., 2015). The pH indicator paper apart from being a standard (Miller et al., 2016), has the advantage of measuring pH values of unbuffered or weakly buffered solutions/samples. Vaginal pH determination by this method is highly accurate (reading accuracy: ±0.1 pH), rapid and reliable. ftp://ftp.mn-net.com/english/Flyer_Catalogs/Test_Sticks_Test_Papers/Fl.%20pH-FixTest_StripsEN.pdf.

ftp://ftp.mn-net.com/english/Instruction_leaflets/Testpapers/pHFix/92130en.pdf.

All clinical samples and measurements were obtained by clinical research staff after written informed consent of participants.

### 2.3 CVF cytokine measurement

The concentrations of 10 cytokines (IL-1α, IL-1β, IL-2, IL-6, IL-8, IL-10, IL-12p70, RANTES, TNF-α and IFN-γ) were determined by a multiplexed bead-based immunoassay, using the BD™ Cytometric Bead Array (CBA) from BD Biosciences, CA, USA. Analysis was carried out according to the BD CBA Human Soluble Protein Master Buffer Kit instruction (http://www.bdbiosciences.com/ds/pm/others/23-13480.pdf), and as previously published (Castillo and Maccallum, 2012; Elshal and Mccoy, 2006). Twenty five microlitres of each sample was pipetted into ELKAY 1.1 ml microtubes and placed onto a 96-well plate. On each plate, lyophilised standards corresponding to each cytokine under investigation were added. Standards for each of the cytokines were combined (pooled) to form a Universal (Top Standard). The Top Standard was then diluted by a factor of 2 until 9 dilutions were created, with the most dilute being 1:256. The first tube (Top standard) contained assay diluent only (no standard dilution) and served as the negative control. This was followed by the preparation of a solution of capture beads, 25 μl of which was added to each tube containing samples and standards. The tube contents were gently mixed and incubated at room temperature for 1 h away from ultraviolet light, allowing adequate binding of the capture antibodies to the cytokines in the samples. After an hour, 25 μl of mixed Phycoerythrin detection reagent was added to the samples and incubated for additional 2 h at room temperature to allow the formation of organometallic compounds (sandwich complexes). The samples were then individually washed and analysed by a BD™ FACSArray Bioanalyzer (Elshal and Mccoy, 2006). Finally, using the FCAP Array v1.0 software, calibration curves were created using the fluorescence data collected from the standard dilutions and median values for each cytokine in the samples were obtained. All cytokine measurements were obtained once, on the same day with the same instruments and by the same operator to ensure uniformity of data.

The concentrations of other proteins were not determined and did not interfere with the concentration of the cytokines under investigation. This is because the assay was optimised to eliminate interference or artefacts from other proteins. The cytokines and anti-cytokine antibodies were tested for cross reactivity with other analytes such as proteins (Elshal and Mccoy, 2006).

### 2.4 Polymerase chain reaction (PCR)

To identify the potential microbial composition/stimulus inducing the expression of cytokines and FFN, the 16S ribosomal DNA of vaginal bacterial species from a randomly selected sample set of 64 (GTP1 = 36, GTP2 = 28) were extracted using the QIAamp DNA mini kit (Qiagen, UK) and amplified by genus/species-specific primers (Sigma-Aldrich, UK) (Bernhard and Field, 2000; Ling et al., 2010; Walter et al., 2002; Zariffard et al., 2002, Supplementary Table S1), to determine the presence of commensal and potentially pathogenic bacterial organisms including *Lactobacillus*, *Gardnerella vaginalis*, *Bacteroides* spp., *Fusobacterium* spp., *Mobiluncus* spp. and *Mycoplasma hominis*. The PCR amplification experiments were performed on an Applied Biosystems 2720 Thermal cycler (Life Technologies, UK) with the following cycling conditions: 95 °C (5 min)—denaturation, followed by 35 cycles of 95 °C (1 min)—denaturing, 50–62 °C depending on the primer sets (1 min)—annealing, 72 °C (1 min)—elongation, with a final extension at 72 °C (7 min). The reaction mix contained 12.5 μl AmpliTaq Gold DNA polymerase (Thermo Fisher Scientific, UK), 5 ng genomic DNA template, 1 μl each of forward and reverse primers (10 μM) in a total reaction volume of 25 μl. The amplification products were visualised on a UV-transilluminator by 1% ethidium bromide-stained agarose gel electrophoresis. Positive results were assigned according to the presence of DNA bands of appropriate sizes.

### 2.5 Statistical analyses

To assess changes in potential genital tract infection/inflammation-associated sPTB biomarkers across the mid-trimester, the ratios of qFFN, cervical length, pH and cytokines measured at 20^+0^–22^+6^ and at 26^+0^–28^+6^ weeks were determined and compared between preterm- and term-delivered women using the Mann-Whitney *U* test. Paired analyses for women who provided samples at both GTPs were performed using Wilcoxon matched-pairs signed rank test. Receiver Operating Characteristics (ROC) curves and binary logit models were also generated to determine their predictive capacity for sPTB. The relationships between maternal clinical data and cytokine expression levels were determined by nonparametric Spearman’s (rho) correlation coefficients. *P-*values <0.05 were considered statistically significant. Bonferroni corrections were applied for multiple measurements. The differences in the prevalence of bacterial sp. between preterm- and term-delivered women, and changes in prevalence of vaginal bacterial species from GTPI to GTP2 ≥25% were also determined by chi-square test. The benchmark of ≥25% was set to buttress any significant differences detected using chi-square test. Analyses were performed on IBM SPSS 22 (IBM Corp., NY, USA), MedCalc 14.8.1 (MedCalc Software bvba, BE) and GraphPad Prism 7.0c (GraphPad Software, Inc. CA, USA) statistical software packages.

## 3 Results

### 3.1 Participants’ demographic and clinical characteristics

The demographic details of the predominantly Caucasian (∼80%) study participants were similar for term- and preterm-delivered cohorts (Table 1, Table 2). Thirty-four participants (72%) whose samples were analysed at GTP1 also had a second sample at GTP2 obtained (i.e. paired samples); and from these, gestational changes in cytokine, qFFN, cervical length and vaginal pH expressed as GTP1/GTP2 ratios were assessed. The cytokines and clinical markers were further analysed as ratios in order to determine any changes in these parameters with increasing gestation, as well as compare their capacity to predict sPTB in serial measurements. Maternal clinical details at both study time points for all study participants are shown in Table 1, while details of women who provided paired samples i.e. provided samples at both GTPs are presented in Table 2. In addition, Supplementary Table 2 shows the number and percentage of women who had a short cervix <25 mm, raised qFFN ≥50 ng/ml, and statistically nonsignificant clinical interventions including cervical cerclage, progesterone, steroids and tocolytics. No BV-positive patient was included in this study and as a result no woman received antibiotics for the treatment of BV. The comparison of cytokine concentrations is indicated in Table 3.

**Table 1:** Maternal demographic and clinical details of all study participants.

Characteristic	GTP1 (20^+0^–22^+6^ weeks)		GTP2 (26^+0^–28^+6^ weeks)	
	Term (n = 25)	Preterm (n = 22)	Term (n = 33)	Preterm (n = 17)
Age, years	30.5 ± 5.6	32.0 ± 4.4	30.1 ± 5.6	31.1 ± 4.9
BMI, kg/m^2^	26.5 ± 4.9	28.7 ± 6.2	27.0 ± 4.6	27.5 ± 5.1
Cervical length, mm	38.0 ± 7.2	29.9 ± 11.0**	32.5 ± 9.1	24.9 ± 13.1
Fetal fibronectin, ng/ml	24.3 ± 32.4	100.2 ± 133	29.7 ± 65.5	63.8 ± 97.6
Vaginal pH	4.2 ± 0.5	4.2 ± 0.5	4.2 ± 0.3	4.3 ± 0.8
GAAS, weeks	21.3 ± 1.1	20.7 ± 1.6	27.0 ± 0.9	27.0 ± 0.9
GAAD, weeks	39.6 ± 1.3	31.9 ± 4.7****	39.5 ± 1.2	33.4 ± 2.8****

*BMI*, body mass index; *GAAD*, gestational age at delivery; *GAAS*, gestational age at sampling; *GTP*, gestational time point. Data presented as Mean ± Standard Deviation (SD).

** *P* < 0.01.

**** *P* < 0.0001.

**Table 2:** Maternal demographic and clinical details of women who provided samples at both gestational time points (paired samples).

	Term, n = 18		Preterm, n = 16	
	GTP1 (20^+0^–22^+6^ weeks)	GTP2 (26^+0^–28^+6^ weeks)	GTP1 (20^+0^–22^+6^ weeks)	GTP2 (26^+0^–28^+6^ weeks)
Age, years	30.3 ± 6.1	30.3 ± 6.1	31.3 ± 4.8	31.3 ± 4.8
BMI, kg/m^2^	27.4 ± 5.3	27.4 ± 5.3	28.3 ± 4.3	28.1 ± 4.7
Cervical length, mm	38.3 ± 5.9	33.6 ± 10.6*	31.8 ± 10.3	25.8 ± 13.0**
Fetal fibronectin, ng/ml	28.8 ± 36.5	12.4 ± 19.4	68.3 ± 101.5	57.7 ± 97.9
Vaginal pH	4.2 ± 0.5	4.1 ± 0.3	4.2 ± 0.6	4.3 ± 0.8
GAAS, weeks	21.3 ± 0.96	26.8 ± 0.73	20.9 ± 1.3	27.0 ± 1.0
GAAD, weeks	39.6 ± 1.2		33.3 ± 2.9****	

*BMI*, body mass index; *GAAD*, gestational age at delivery; *GAAS*, gestational age at sampling; *GTP*, gestational time point. Data presented as Mean ± Standard Deviation (SD).

* *P* < 0.05.

** *P* = 0.01.

**** *P* < 0.0001.

**Table 3:** Comparison of cervicovaginal fluid cytokine concentrations (pg/ml) in relation to delivery outcome.

Outcome	IL-1α	IL-1β	IL-2	IL-6	IL-8	IL-10	IL-12	RANTES	TNF-α	IFN-γ
GTP1 (20–22 weeks)										
Term (n = 25)	1167 (375.6–4053)	773.8 (285.6–2351)	24.4 (16.8–29.0)	13.6 (4.9–42.4)	9771 (3437–15864)	6.5 (5.3–9.0)	10.3 (4.7–13.7)	9.2 (6.2–12.9)	6.6 (4.6–8.3)	15.9 (4.2–19.6)
Preterm (n = 22)	812.9 (216.5–1521)	590.8 (132.6–1121)	23.1 (18.3–26.2)	9.9 (5.6–22.3)	6873 (386.4–14548)	6.2 (5.7–8.4)	10.2 (4.9–12.1)	6.7 (4.8–10.6)	6.4 (4.8–7.5)	14.9 (4.3–18.9)
*P*-value	0.22	0.40	0.54	0.46	0.42	0.93	0.50	0.22	0.47	0.63

GTP2 (26–28 weeks)										
Term (n = 33)	661.3 (270.3–1576)	566.4 (59.3–1438)	0.0 (0.0–6.1)	7.3 (0.0–22.4)	6979 (2596–13990)	0.0 (0.0–1.4)	1.6 (0.0–2.1)	3.4 (1.9–7.8)	0.0 (0.0–1.3)	0.0 (0.0–0.0)
Preterm (n = 17)	888.3 (549.8–2579)	659 (310.6–1152)	0.0 (0.0–6.3)	15.6 (2.9–290.5)	10225 (4815–15493)	0.0 (0.0–1.3)	0.0 (0.0–2.0)	4.8 (1.1–8.2)	0.0 (0.0–1.3)	0.0 (0.0–0.0)
*P*-value	0.24	0.43	0.47	0.09	0.39	0.71	0.08	0.81	0.94	NA

GTP1/GTP2 ratio										
Term	1.59 (0.2–2.9)	3.1 (0.3–14.9)	4.0 (2.8–4.8)	2.2 (0.4–5.4)	1.0 (0.1–3.4)	4.0 (2.9–7.3)	4.7 (2.6–6.1)	2.2 (1.7–4.6)	5.8 (3.0–6.2)	4.8 (4.8–4.8)
(n = 18)	(n = 17)	(n = 17)	(n = 9)	(n = 11)	(n = 17)	(n = 9)	(n = 12)	(n = 13)	(n = 7)	(n = 1)
Preterm	0.6 (0.01–1.5)	0.6 (0.1–1.9)	4.0 (1.8–9.3)	0.6 (0.03–3.38)	0.3 (0.04–1.4)	5.6 (4.2–4474)	3.6 (2.4–5.7)	1.3 (0.5–1.5)	4.7 (2.5–5.5)	6.5 (6.5–6.5)
(n = 16)	(n = 16)	(n = 16)	(n = 8)	(n = 15)	(n = 16)	(n = 5)	(n = 5)	(n = 13)	(n = 5)	(n = 1)
*P*-value	0.25	0.04^*^	0.96	0.09	0.14	0.19	0.65	0.004^**^	0.34	NA

*GTP*, gestational time point; *NA*, not applicable. Data presented as median (25th and 75th percentiles). GTP1/GTP2 ratios were computed for only those women who provided paired samples i.e. women sampled at both gestational time points. The total n = 34 (Term = 18 & Preterm = 16) are those women who were sampled at both GTPs. Samples without a particular cytokine or cytokine concentration below the detectable limit of the assay kit (indicated as a value of zero) were subsequently omitted when computing ratios.

All women (i.e. term- and preterm-delivered) included in this study experienced spontaneous labour before delivery. Cases of emergency and elective iatrogenic non-spontaneous deliveries (inductions and Caesarean sections), and late miscarriages were excluded.

### 3.2 Cytokine expression levels

**Fig. 1:**
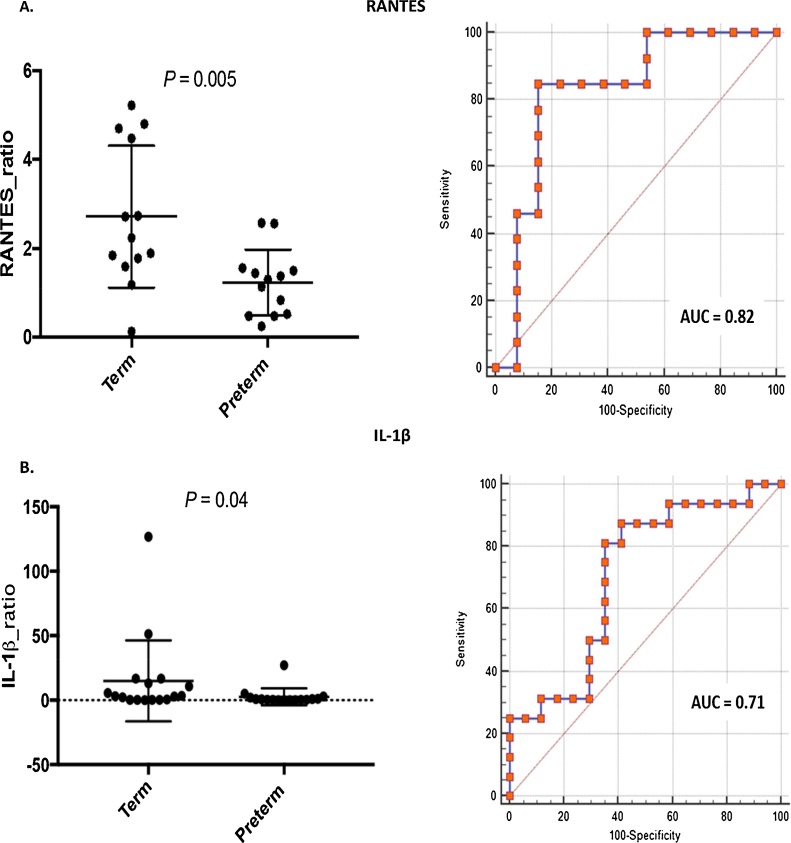
Predictive value of mid-trimester cervicovaginal fluid cytokines for PTB expressed as ratios between 20^+0^–22^+6^ and 26^+0^–28^+6^ weeks. **A.** RANTES (n = 26), **B.** IL-1β (n = 33), analysed per-patient. All data (i.e. both term and preterm women) were included in the analyses. *AUC*, area under the ROC curve, *Sen*, sensitivity, *Spec*, specificity.

As summarised in Table 3, none of the cytokines at both 20^+0^–22^+6^ weeks (GTP1) and 26^+0^–28^+6^ weeks (GTP2) differed significantly between term- and preterm-delivered women. However, when expressed as a ratio between GTP1 and GTP2 for those women with paired samples, IL-1β (*P <* 0.05) and RANTES (*P <* 0.01) differed between term-delivered and preterm-delivered women (Fig. 1), consistent with a higher rate of decrease in their mean expression levels, with increasing gestation, in the term-delivered cohort compared to the preterm-delivered women. Also, tracking the changes in individual cytokines using line plots (Supplementary Fig. 2), showed a gestation-associated significant decrease in IL-2, IL-10, IL-12, RANTES, and TNF-α in the term-delivered women; while only IL-2, IL-10, IL-12 and TNF-α decreased in a similar manner in the preterm-delivered women.

Table 4 summarises the predictive performance of significantly predictive cytokine biomarkers for sPTB at the two study gestations (IL-1β, RANTES), singly, expressed as ratios and in combination. GTP1/GTP2 ratios of other studied cytokines did not differ significantly between term- and preterm-delivered women and were not predictive of sPTB.

**Table 4:** Predictive performance of biomarkers and their ratios at the two study gestations for preterm birth <37 weeks – Area under the ROC curves of Sensitivity vs. 1- Specificity.

Biomarkers	AUC (95% CI)	Sen	Spec	PPV	NPV	LR+	LR-	*P*-value
**RANTES ratio**	**0.82 (0.62–0.94)**	**85**	**85**	**85**	**85**	**5.5**	**0.2**	**0.0003**
GTP1	0.61 (0.44–0.77)							0.22
GTP2	0.52 (0.35–0.769)							0.80
**IL 1β ratio**	**0.71 (0.53–0.85)**	**81**	**65**	**68**	**79**	**2.3**	**0.3**	**0.02**
GTP1	0.57 (0.41–0.74)							0.40
GTP2	0.57 (0.41–0.73)							0.42
**qFFN ratio**	**0.64 (0.45–0.80)**	**47**	**94**	**88**	**67**	**7.9**	**0.6**	**0.17**
GTP1	0.67 (0.50–0.84)							0.06
GTP2	0.67 (0.50–0.84)							0.06
**CL ratio**	**0.58 (0.40–0.75)**	**63**	**72**	**67**	**68**	**2.3**	**0.5**	**0.42**
GTP1	0.75 (0.60–0.90)							0.005
GTP2	0.64 (0.46–0.83)							0.11
**pH ratio**	**0.58 (0.40–0.75)**	**38**	**94**	**86**	**63**	**6.8**	**0.7**	**0.42**
GTP1	0.53 (0.36–0.70)							0.74
GTP2	0.53 (0.35–0.71)							0.71

Combined ROC curves of GTP1/GTP2 ratios of biomarkers								
IL-β ratio + RANTES ratio	0.84 (0.64–0.95)	77	92	91	80	10	0.3	0.0001
IL-β ratio + qFFN ratio	0.70 (0.51–0.85)	93	44	61	88	1.7	0.2	0.04
RANTES ratio + qFFN ratio	0.82 (0.61–0.95)	83	83	83	83	5	0.2	0.0007
IL-β ratio + RANTES ratio + qFFN ratio	0.83 (0.63–0.95)	75	92	90	79	9	0.3	0.0003

All data (i.e. both term and preterm women) were included in the analyses. *AUC*, area under the ROC curve; *CI*, confidence interval; *CL*, cervical length, *qFFN*, quantitative fetal fibronectin; *PPV*, positive predictive value; *NPV*, negative predictive value; *LR+*, positive likelihood ratio; *LR-*, negative likelihood ratio; *Sen*, sensitivity; *Spec*, specificity; *GTP*, gestational time point; *GTP1*, 20^+0^-22^+6^ weeks; *GTP2*, 26^+0^-28^+6^ weeks; *Ratio*, GTP1/GTP2.

### 3.3 Quantitative fetal fibronectin, cervical length and vaginal pH

The preterm-delivered women had significantly shorter cervical lengths compared to the term-delivered women at GTP1 (20^+0^–22^+6^ weeks) only in the unpaired cohort (Table 1). Also, among women with paired samples, cervical length significantly decreased with increasing gestation in both term- and preterm-delivered women (Table 2 and Supplementary Fig. 1). The FFN concentrations and vaginal pH values were not significantly different at any of the study time points and among the paired samples (Table 1, Table 2). Furthermore, per-patient analysis of the change in FFN concentrations, cervical length, and vaginal pH between GTP1 and GTP2, expressed as ratios, did not differ between term- and preterm-delivered women. These biomarkers were not predictive of sPTB either singly or when expressed as GTP ratios except cervical length at GTP1 (Table 4).

### 3.4 Combination of cytokine levels with quantitative fetal fibronectin, cervical length and vaginal pH

**Fig. 2:**
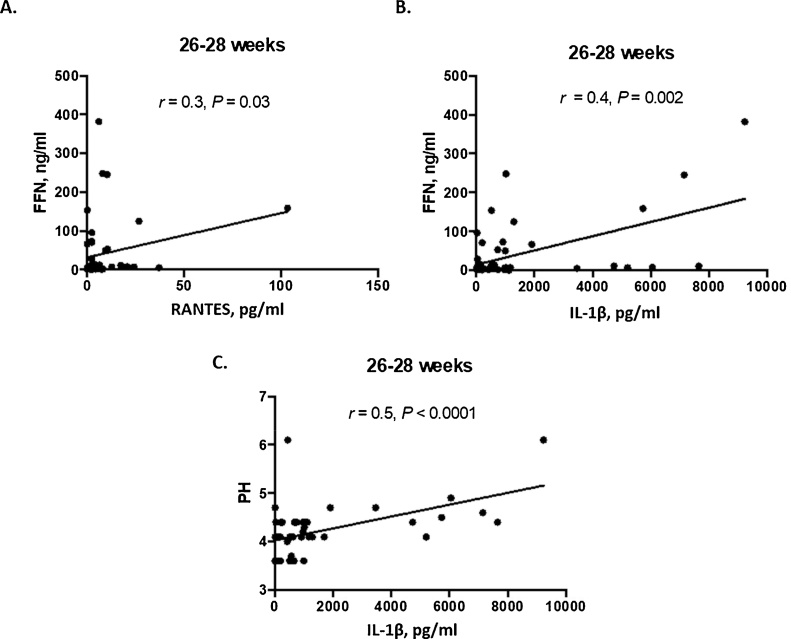
Association of common clinical assessment markers of PTB and CVF cytokines at late mid-trimester (26^+0^–28^+6^ weeks). Correlation of fetal fibronectin (FFN), IL-1β, and RANTES concentrations, n = 47 (**A**–**B**); and correlation of vaginal pH and IL-1β, n = 49 (**C**), for both study cohorts.

**Fig. 3:**
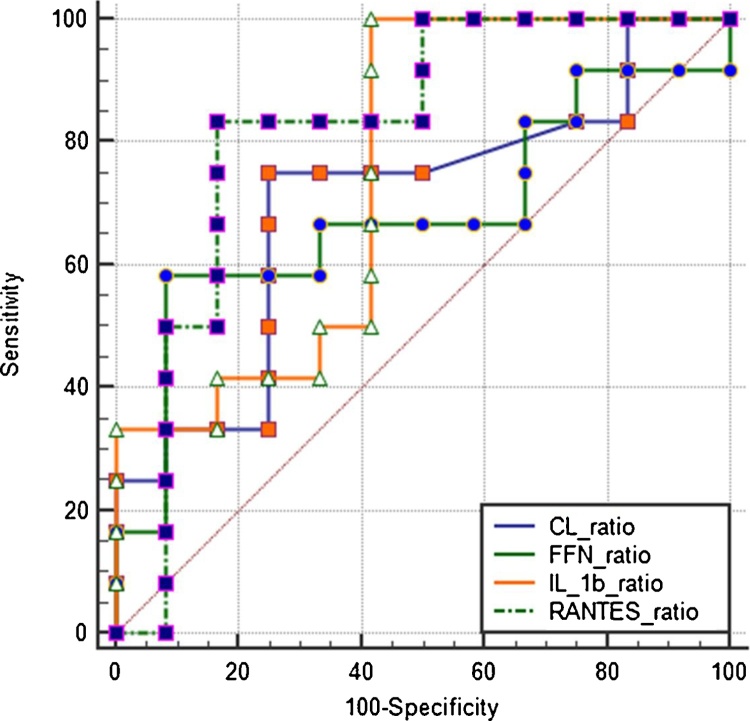
Comparison of ROC curves for prediction of PTB: 20^+0^–22^+6^/26^+0^–28^+6^ weeks ratios of RANTES (broken green), IL-1β (orange), quantitative FFN (continuous green), and CL (blue). The change in concentration of CVF RANTES between 20^+0^–22^+6^ and 26^+0^–28^+6^ weeks expressed as a ratio indicated the highest predictive capacity for spontaneous PTB. These comparisons were made for only those patients (term and preterm) that had values for all five biomarkers. (For interpretation of the references to colour in this figure legend, the reader is referred to the web version of this article.) *CL*, ultrasound cervical length; *FFN*, fetal fibronectin.

For both term- and preterm-delivered cohorts, at 26^+0^–28^+6^ weeks (GTP2), qFFN correlated with RANTES (*r* = 0.3, P = 0.03) and IL-1β (*r* = 0.4, *P* = 0.002), while the vaginal pH only correlated with IL-1β (*r* = 0.5, *P <* 0.0001) (Fig. 2). No correlations were observed between qFFN, vaginal pH and cytokine concentrations measured at 20^+0^–22^+6^ weeks (GTP1). Furthermore, combining ratios of RANTES and qFFN, RANTES and IL-1β, IL-1β and qFFN, as well as RANTES, IL-1β and qFFN by binary logit regression models did not significantly improve the predictive capacity of the ratio of RANTES alone for sPTB (Table 4 and Fig. 3).

### 3.5 Prevalence of vaginal anaerobic bacterial species at study gestations

**Fig. 4:**
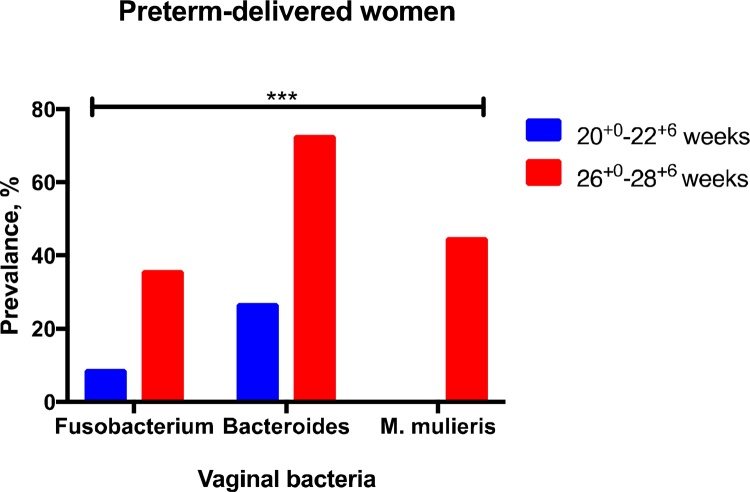
Increase in the prevalence of pathogenic vaginal bacterial species among the preterm-delivered women with advancing gestation. **P < 0.001.

Representative pictures of bacterial genomic DNA bands visualised on a UV-transilluminator by agarose gel electrophoresis are shown in Supplementary Fig. 3. In the preterm-delivered women, there was an increased prevalence of vaginal anaerobic bacterial species between GTP1 and GTP2 groups, particularly the *Fusobacterium*, *Bacteroides* and *Mobiluncus mulieris* species (*P* = 0.0006) (Table 5 and Fig. 4). However, this increase was not associated with changes in individual cytokine concentrations, qFFN and cervical length. Interestingly, the preterm-delivered women with *Bacteroides*-positive vaginal specimens had increased vaginal pH (expressed as lower ratio) with advancing gestation from 20^+0^–22^+6^ to 26^+0^–28^+6^ weeks, compared to other preterm-delivered women with *Bacteroides-*negative samples (pH ratio: 0.95 ± 0.08 vs. 1.2 ± 0.09, *P* = 0.02).

**Table 5:** Prevalence of endogenous vaginal bacterial species identified by PCR.

Bacterial sp., %	GTP1 (20^+0^–22^+6^ weeks)		GTP2 (26^+0^–28^+6^ weeks)	
	Term (n = 25)	Preterm (n = 11)	Term (n = 17)	Preterm (n = 11)
*Lactobacillus* sp.^a^	100	100	100	100
*Fusobacterium* sp.^b^	20	9	12	36*
*Bacteroides-Prevotella*^b^	16	27	24	73*
*Mobiluncus mulieris*^b^	4	0	6	45*
*Gardnerella vaginalis*^b^	92	100	82	73
*Mobiluncus curtisii*^b^	28	36	18	27
*Mycoplasma hominis*^b^	4	18	0	9

*GTP*, gestational time point.

a Commensal/protective bacterial sp.

b Anaerobic bacterial sp. associated with abnormal vaginal microflora, infection and PTB.

* Increase in the prevalence of bacterial sp. (≥25%, *P <* 0.001) in the preterm-delivered women from 20^+0^–22^+6^ to 26^+0^–28^+6^ weeks.

*Lactobacillus* sp., the predominant commensal organism in the vaginal microenvironment was identified in all study participants, while prevalence of *Gardnerella vaginalis*, *Mobiluncus curtisii* and *Mycoplasma hominis* did not change significantly within and between the groups (Table 5).

## 4 Discussion

There is substantial evidence linking bacterial colonisation of the female reproductive tract and ascending intrauterine infection with inflammation-associated PTB (Agrawal and Hirsch, 2012; Aldunate et al., 2015; Chan, 2014; Genc et al., 2004; Goldenberg et al., 2005; Menon and Fortunato, 2007; Witkin et al., 2011). In this study, we sought to determine the changes in mid-trimester CVF cytokine concentrations in women at risk of spontaneous PTB (sPTB). We hypothesised that the changes between the two study time points could differ between the women who delivered preterm and those who delivered at term, reflecting altered processes associated with imminent inflammation-associated PTB, and that these differences may be predictive of sPTB. We observed that although none of the cytokines was predictive of sPTB at either study gestations, women who delivered at term demonstrated greater decrease in levels of IL-1β and RANTES from 20^+0^–22^+6^ to 26^+0^–28^+6^ weeks gestation than their preterm-delivered counterparts, expressed as a ratio between the two study gestations. We also observed that the RANTES ratio between the study gestations predicted spontaneous labour and delivery before 37 weeks better than the ratios of IL-1β, FFN and cervical length, and that combining these markers did not improve prediction of sPTB.

Of the pro-inflammatory cytokines and chemokines studied, only the 20^+0^–22^+6^/26^+0^–28^+6^ weeks ratios of RANTES and IL-1β were predictive of sPTB. In women who delivered preterm and studied at both gestations, RANTES levels remained fairly unchanged between 20^+0^–22^+6^ and 26^+0^–28^+6^ weeks, whilst it decreased (about 3-fold) in their term-delivered counterparts. For IL-1β, the term-delivered women experienced a decrease, while the preterm women showed an increase with advancing gestation. These observations suggest that subclinical inflammation and secretion of cytokines into CVF may be triggered several weeks prior to the onset of sPTB. If confirmed in larger studies, serial assessment of cervicovaginal RANTES and IL-1β expression concentrations in mid-trimester may prove a marker for sPTB.

We investigated CVF cytokine concentrations because the inflammatory processes associated with PTB (Agrawal and Hirsch, 2012; Romero et al., 2005; Tency, 2014) include the elaboration of cytokines in gestational and cervical tissues (Vrachnis et al., 2012). Cytokine-mediated stimulation of prostaglandin (PG) and matrix metalloproteinase (MMP) enzyme weakens cervical and amniotic membrane barriers, facilitating microbial invasion of the amniotic cavity, myometrial activation, rupture of membranes and birth (Agrawal and Hirsch, 2012; Kalinka et al., 2005; Oh et al., 2017; Park et al., 2016; Vrachnis et al., 2012; Witkin et al., 2013). For instance, TNF-α stimulates the production of IL-6, IL-8, PGs and MMPs. IL-6 stimulates antibody production and activates the acute phase response, whilst IL-8 stimulates neutrophil degranulation. Similarly, IL-1 stimulates production of collagenases, elastases and PGs (Kalinka et al., 2005; Vrachnis et al., 2012). Whilst these pro-inflammatory cytokines play a role in triggering PTL, IL-10, an anti-inflammatory cytokine, appears crucial for maintaining uterine quiescence (Nenadić and Pavlović, 2008; Vrachnis et al., 2012) by supressing the production of IL-1, IL-6, TNF-α and PGs (Vrachnis et al., 2012).

Although at the outset we speculated that women who deliver preterm will have significantly higher CVF cytokine concentrations in mid-trimester than their counterparts who delivered at term, it has also been reported that host immune hypo-responsiveness associated with reduced levels of pro-inflammatory cytokines in CVF may permit ascending infection, chorioamnionitis (Simhan et al., 2003), and PTL. In one study, women with pathogenic genital microflora demonstrated low levels of CVF IL-1α, 1L-1β, IL-6, and IL-8 at mid-gestation and experienced higher PTB rates (Kalinka et al., 2005), and elevated amniotic fluid IL-6 and Interferon gamma-inducible protein (IP)-10 concentrations in preterm-delivered women without an identifiable bacterial cause have been observed (Gervasi et al., 2012). How CVF cytokine expression levels alter with delivery gestation is still unclear. Dysregulation of an optimum immunological balance (Guaschino et al., 2006) may alter CVF cytokine concentrations leading to PTB (Romero et al., 2007). Such dysregulation may result from uncontrolled subclinical proliferation and ascent of anaerobes and a depletion of protective *Lactobacillus*, leading to NF-κB-induced secretion of inflammatory cytokines (Aldunate et al., 2015), PTL and delivery (Holst and Garnier, 2008; Menon et al., 2006; Tency, 2014). We have noted increased or unchanged expression levels of 1L-1β and RANTES, as well as a trend towards decreased IL-10, with advancing gestation in our preterm-delivered cohort, consistent with the above thesis and in support of a putative role for inflammation in sPTB.

RANTES or chemokine (C-C motif) ligand 5 (CCL5) is produced by immune, endothelial and gestational tissues. Being a potent chemoattractant and inducer of inflammatory cells, a role in parturition and regulation of host immune response to genital infection has been suggested (Athayde et al., 1999; Hamilton et al., 2013; Vrachnis et al., 2012). Recently, significantly higher blood RANTES levels were observed in women in PTL (Hamilton et al., 2013). Maternal serum RANTES and IL-10 levels together with cervical length appear predictive of delivery within 7 days of assessment in women with threatened PTL (Tsiartas et al., 2012). Similarly, IL-1β produced by polymorphonuclear leukocytes (Vrachnis et al., 2012), appears to be a major effector of infection-associated PTB (Genc et al., 2004; Sadowsky et al., 2006), with elevated levels identified in amniotic fluid of women diagnosed with amniotic infection (Romero et al., 1992a, Romero et al., 1992b; Romero et al., 2015). It appears to trigger PTL by stimulating apoptosis and up-regulating MMPs, PGs (Menon and Fortunato, 2007; Romero et al., 1992a, Romero et al., 1992b; Sadowsky et al., 2006), as well as IL-6, IL-8, and TNF-α (Genc et al., 2004). Our findings of a higher rise in CVF IL-1β concentrations in women who delivered preterm and a decrease in RANTES in the term women would seem consistent with their roles in the inflammatory signalling associated with sPTB. These changes in cytokine expression were observed despite treatment of a negligible proportion of women with progesterone, cerclage and other common interventions. This is not surprising as progesterone administration may not significantly influence the concentration of CVF cytokines (Chandiramani et al., 2012).

We also compared the predictive capacities of changes in RANTES and IL-1β expression levels across the mid-trimester with those of screening tests used in clinical practice—CVF FFN, ultrasound cervical length and vaginal pH. Spontaneous PTB was associated with shorter cervical length at 20^+0^–22^+6^ weeks and cervical length decreased with increasing gestation. Though FFN level was not associated with sPTB, it correlated with RANTES and IL-1β levels at the later study gestation. This observations could be supported by the established link between inflammation, chorioamnionitis, membrane activation, leakage and detection of FFN in CVF, cervical remodelling and subsequent myometrial activation (Agrawal and Hirsch, 2012; Berghella et al., 2008; Georgiou et al., 2015; Goepfert et al., 2000; Holst et al., 2006; O’Hara et al., 2013).

We also qualitatively examined the bacterial composition of the vaginal microbiota of women without clinical infection, to identify potential microbial stimuli for the cytokine expression profiles that we observed at mid-gestation. In the women who delivered prematurely, selected anaerobes (*Bacteroides* spp. *Fusobacterium* spp., and *M. mulieris*) became more prevalent at the later study gestation, compared to term-delivered women. It is plausible that this change in microbial floral densities may contribute to the altered inflammatory cytokine concentrations (especially RANTES and IL-1β) at the later study gestation in the preterm-delivered group of women. There was no significant difference in the prevalence of *G. vaginalis* and *M. curtisii* between term- and preterm-delivered women and between the study time points. This supports reports that *G. vaginalis* is not uniquely related to BV or delivery outcome (Fredricks et al., 2005; Fredricks et al., 2007; Hillier et al., 1995; Vitali et al., 2015), being found in nearly all healthy women (Zozaya-Hinchliffe et al., 2010). Taken together, our observations lend credence to a possible association between female genital tract colonisation by anaerobic bacteria, and infection/inflammation-associated sPTB (Ramos et al., 2015; Romero et al., 2007; Taylor et al., 2013; Witkin et al., 2011). Though *M. hominis,* a member of the class Mollicutes, did not differ in relation to delivery outcome in this study, another member of the same class not investigated in the current study*—Ureaplasma—*appears implicated in intra-amniotic infection and PTB, along with *M. hominis* (Keelan et al., 2016; Oh et al., 2017), and warrants further study.

Our study has several limitations. Firstly, the study sample sizes are small and may be underpowered to detect subtle cytokine associations with sPTB. Secondly, we have observed changes at limited gestational time points and can draw no conclusions regarding the gestations at which these changes commence, neither can we state whether they continue into the third trimester. Thirdly, relationship between individual bacterial load (quantities) and cytokine concentrations by quantitative PCR was not performed and is crucial for future investigation.

In conclusion, we report that subsequent spontaneous preterm birth appears associated with a lesser rate of decline of CVF expression levels of RANTES and IL-1β in mid-trimester of pregnancy. Further, sufficiently powered, studies are required to confirm these observations, determine the microbial and other mechanisms involved, and elucidate whether CVF cytokine assays may have future predictive clinical utility for sPTB.

## Funding

This work was supported by the Medical Research Council, UK (grant number: MR/J014788/1); and EA was supported with a Ph.D. studentship from the Niger Delta Development Commission and Bayelsa State Scholarship Board of Nigeria.
