# Supplementary material for: Mid-gestational changes in cervicovaginal fluid cytokine levels in asymptomatic pregnant women are predictive markers of inflammation-associated spontaneous preterm birth
Source: J Reprod Immunol. 2018 Apr;126:1–10. doi: 10.1016/j.jri.2018.01.001 (PMC5886036; doi:10.1016/j.jri.2018.01.001)

**Supplementary Figure 1.** Change in ultrasound cervical length (CL) and quantitative fetal fibronectin (qFFN) with advancing gestation. Paired analysis included only women who provided samples at both gestational time points i.e. 20^+0^-22^+6^ and 26^+0^-28^+6^ weeks.

**Supplementary Figure 2.** Change in cervicovaginal fluid cytokine concentrations with increasing gestation. Paired analysis included only women who provided samples at both gestational time points i.e. 20^+0^-22^+6^ and 26^+0^-28^+6^ weeks. Note that samples without a particular cytokine, or cytokine concentration below the detectable limit of the assay kit (indicated as a value of zero), were omitted when computing differences using Wilcoxon matched-pairs signed rank test.

**Supplementary Figure 3.** Representative pictures of bacterial genomic DNA bands visualised on a UV-transilluminator by agarose gel electrophoresis: 1) *Lactobacillus* sp., 2) *L. jensenii*, 3) *L. jensenii* and/or *L. crispatus*, 4) *Fusobacterium* sp., 5) *G. vaginalis*, 6) *Bacteroides-Prevotella* sp., 7) *Mobiluncus cutisii*, 8) *Mycoplasma hominis*. Bacterial DNA standards used as positive controls: a) *Fusobacterium nucleatum* (ATCC 25586), b) *Gardnerella vaginalis* (NCTC 11292), c) *Bacteroides fragilis* (NCTC 9343), d) Streptococcus agalactiaea (clinical isolate), e) *Mobiluncus curtisii* (NCTC 11656).

**Supplementary Table 1**: Primer sequences used for bacterial 16S rDNA amplification

| **Primer** | **Sequence (5’-3’)** | **Target bacterial sp.** | **Annealing temp (ºC)** | **Amplicon**  **size**  **(bp)** | **Reference** |
| --- | --- | --- | --- | --- | --- |
| LABF  LABR | AGAGTTTGATYMTGGCTCAG  CACCGCTACACATGGAG | *Lactobacillus* | 62 | 667 | ([Ling et al., 2010](#_ENREF_36)) |
| FBF  FBR | ACTCCTACGGGAGGCAGCAGT  CGAATTTCACCTCTACACTTGT | *Fusobacterium* | 60 | 341 | ([Walter et al., 2002](#_ENREF_60)) |
| GV1F  GV3R | GGAAACGGGTGGTAATGCTGG  CGAAGCCTAGGTGGGCCATT | *G. vaginalis* | 65 | 125 | ([Zozaya-Hinchliffe et al., 2010](#_ENREF_64)) |
| BAC32F  BAC708R | AACGCTAGCTACAGGCTT  CAATCGGAGTTCTTCGTG | *Bacteroides-Prevotella* | 53 | 676 | ([Bernhard and Field, 2000](#_ENREF_6)) |
| M. curt-440F  M. curt-1026R | TTCTCGCGAAAAAGGCACAG  CTGGCCCATCTCTGGAACCA | *M. curtisii* | 57 | 586 | ([Fredricks et al., 2007](#_ENREF_14)) |
| Mobil-577F  M.mulie-1026R | GCTCGTAGGTGGTTCGTCGC  CCACACCATCTCTGGCATG | *M. mulieris* | 62 | 449 | ([Fredricks et al., 2007](#_ENREF_14)) |
| Mh1-F  Mh2-R | CAATGGCTAATGCCGGATACGC  GGTACCGTCAGTCTGCAAT | *M. hominis* | 62 | 334 | ([Zariffard et al., 2002](#_ENREF_63)) |

**Supplementary Table 2**: Proportion of women with short cervix, raised fetal fibronectin and interventions for preterm delivery

|  | GTP1 (20^+0^-22^+6^ weeks) | | GTP2 (26^+0^-28^+6^ weeks) | |
| --- | --- | --- | --- | --- |
|  | Term, n = 25 | Preterm, n = 22 | Term, n = 33 | Preterm, n = 17 |
| Cervical length  < 25 mm | 1 (4) | 7 (32) | 5 (15) | 9 (53) |
| Fetal fibronectin  ≥ 50 ng/ml | 3 (12) | 10 (45) | 5 (15) | 7 (41) |
| Cervical cerclage | 0 | 2 (9) | 0 | 2 (12) |
| Progesterone | 1 (4) | 2 (9) | 2 (6) | 5 (29) |
| Steroids | 0 | 1 (4.5) | 1 (3) | 3 (18) |
| Tocolytics | 0 | 1 (4.5) | 0 | 1 (6) |

Data presented as number and percentage of women, n (%). No woman received antibiotics for the treatment of bacterial vaginosis (BV) because BV-positive patients were excluded from this study. *GTP*, gestational time point.

**Supplementary Figure 1.**


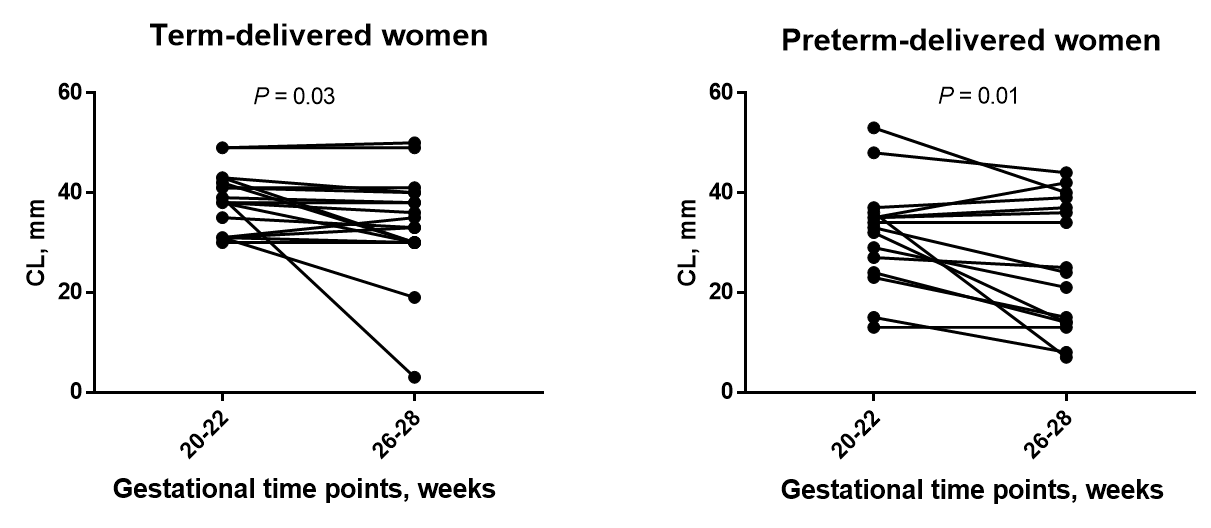


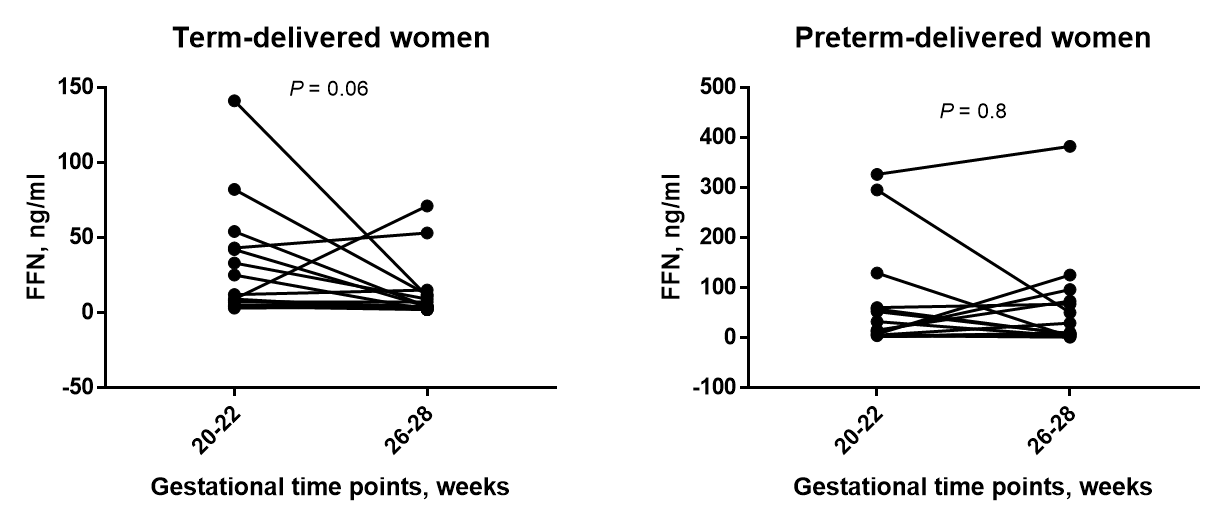


**Supplementary Figure 2.**

**A.**


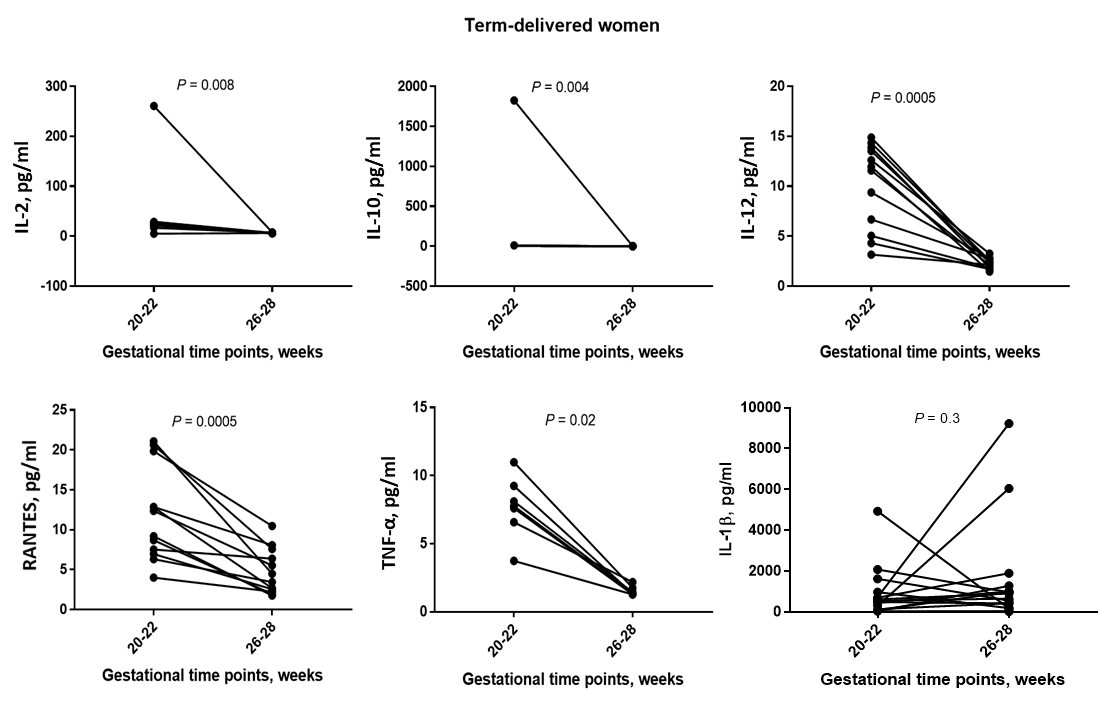


**B.**


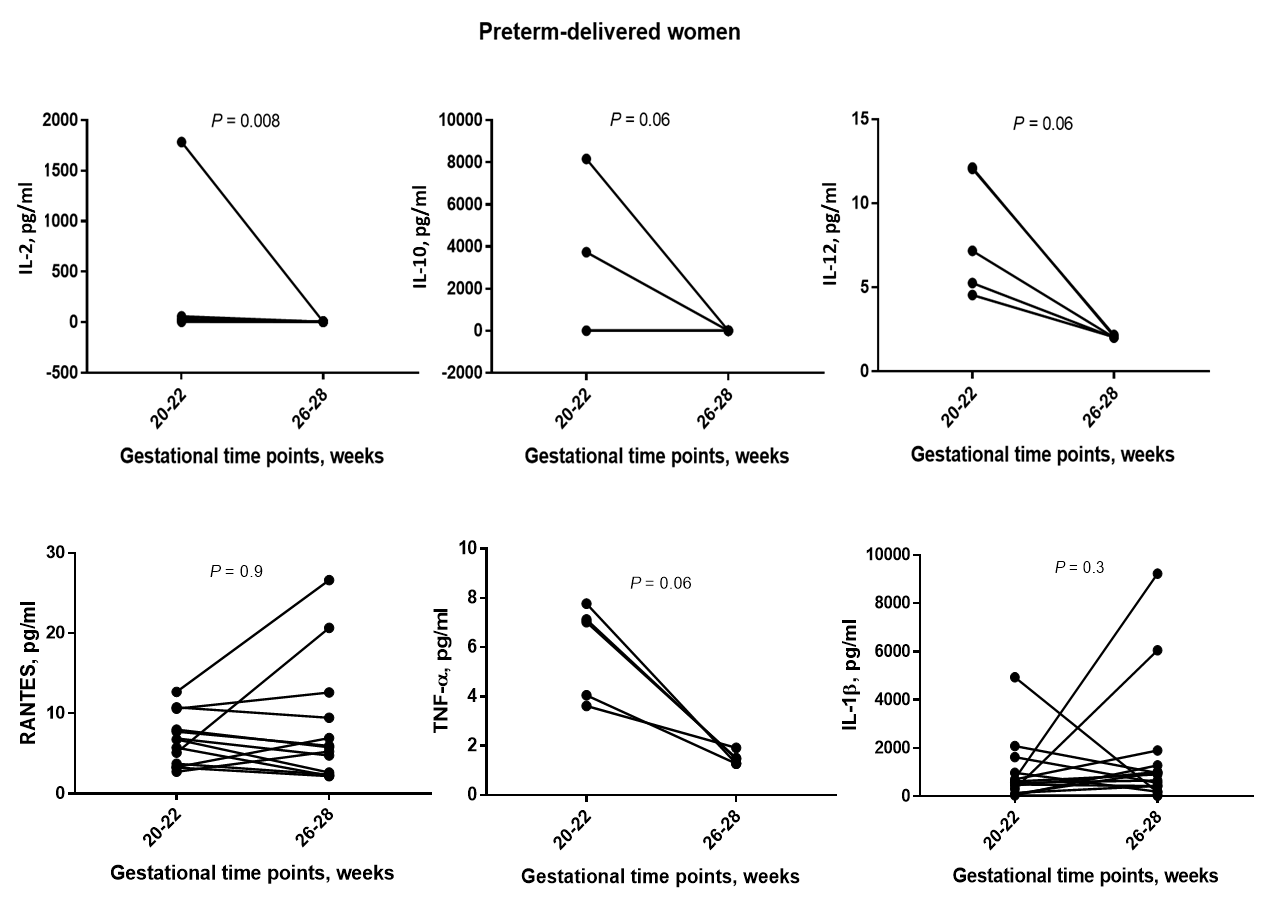


**Supplementary Figure 3.**


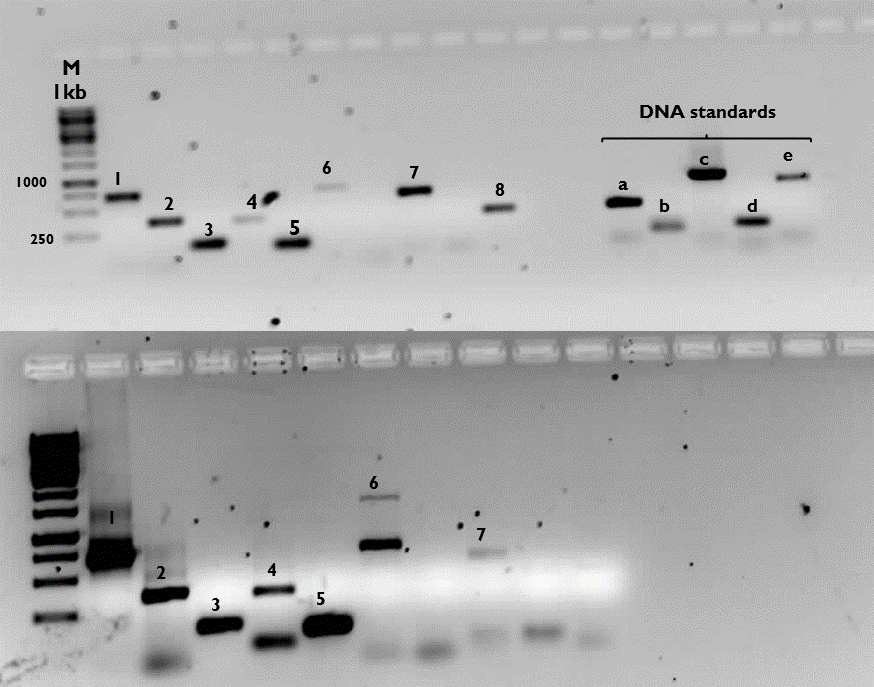

Supplement: Supplementary file 1 [file mmc1.docx]
